# Injury of the Infrapatellar Branch of Saphenous Nerve Between Vertical and Oblique Skin Incision in Medial Opening Wedge High Tibial Osteotomy

**DOI:** 10.5704/MOJ.2307.009

**Published:** 2023-07

**Authors:** W Kongcharoensombat, P Charoensri

**Affiliations:** 1 Department of Orthopaedics, Lerdsin Hospital, Bangkok, Thailand; 2 Department of Orthopaedics, Sunpasitthiprasong Hospital, Ubon Ratchathani, Thailand

**Keywords:** infrapatellar nerve, vertical incision, oblique incision, HTO

## Abstract

**Introduction:**

The infrapatellar branch of saphenous nerve (IPBSN) has anatomic variations and prone to injury during surgery around the medial side of the knee. High tibial osteotomy is one of the procedures that may be risky to the IPBSN. This research was aimed to establish which skin incision (vertical vs oblique) is less likely to damage to the IPBSN and also to study the anatomy of the IPBSN, with the institutional review board reference (No. LH611054, date 10/1/2020). The primary outcomes are aimed to establish which skin incision (vertical vs oblique) is less damaging to the IPBSN. The secondary outcome is to study about the anatomy of the IPBSN.

**Materials and methods:**

Twenty-two fresh cadavers (forty-four knees) were dissected by randomisation under the block of four technique, and two different incisions were performed for each knee. Exploration was performed from the skin incision to the IPBSN around the incision zone. If the discontinuity of the nerve was found, it was classified as IPBSN injury. The anatomic measurement was performed. The IPBSN injury between two groups were analysed with the chi-square test.

**Results:**

The risk of IPBSN injury in the oblique group was 2 from 22 knees (9.1%), and 12 knees from 22 knees (54.5%) in the vertical group (P=0.001). Most common number of branch(es) found, is one branch, the horizontal distance ranged from 2.6cm to 8.5cm (average 5.7±1.6), the vertical distance ranged from 4.4cm to 12.6cm (average 7.6±1.9) and the declination angle ranged from 6° to 87° (average 34.7±24.3).

**Conclusion:**

The risk of the IPBSN injury in oblique skin incision may be less than the vertical incision in the medial opening wedge HTO.

## Introduction

The infrapatellar branch of the saphenous nerve (IPBSN) is the cutaneous nerve which pierces the sartorius and deep fascia; which curves, downward and forwards to supply the skin over the anteromedial aspect of the knee. The anatomy of IPBSN varies from individual to individual such as branches, position or decline angle. Due to the variations and small size of the nerve, it may be damaged commonly during medial approaches to knee surgery. Complications resulting from the nerve injury may lead to a various degree of discomfort, chronic pain, altered sensation and irritating paraesthesia^1-7^.

High tibial osteotomy (HTO) is one of the procedures to treat medial compartment osteoarthritis of knee, by shifting the weight bearing from the medial to the lateral compartment of knee^8^. In this research, we focus on the medial opening wedge high tibial osteotomy in the incision located on the medial side below the knee joint line. The position of the incision may cause a risky injury to the IPBSN. Currently, two incisions are favourable. The vertical incision is the conventional method. The oblique incision is used in the minimally invasive technique. Both incisions are associated with the IPBSN injury during the incision from skin down to bone.

## Materials and Methods

**Fig 1:**
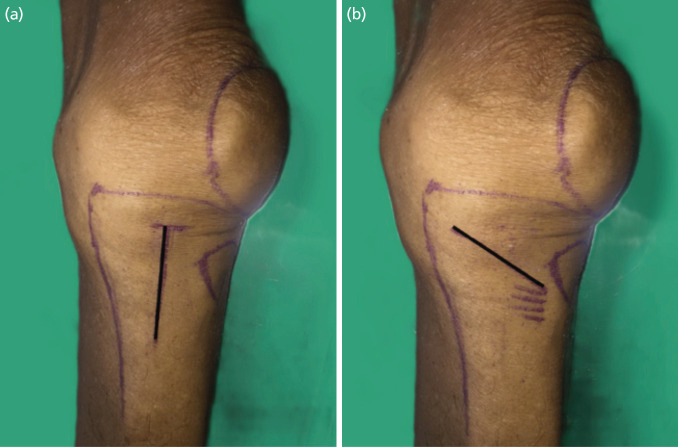
(a) Black line is the vertical incision, starting 1cm below the joint line. (b) Black line is the oblique incision which was made from the superior to the pes anserinus insertion to the most medial point of the knee joint.

Twenty-two fresh cadavers (forty-four knees) were sourced from the department of anatomy. They were dissected by randomisation under the block of four techniques. We used a computer-generated random sequence to obtain the block of four which was used for determining which side would undergo vertical or oblique incision. We set the protocol so that both sides would be randomised using the vertical or oblique incisions. Each of the two groups, vertical and oblique incisions, comprised twenty-two knees per group. We excluded cadavers which had a surgical scar around the area of examination. A 5cm vertical incision was made centrally between the medial aspect of the tibial tuberosity and the medial aspect of the tibia. It was started 1cm below the joint line as shown (Fig. 1a). A 5cm oblique incision was made superior to the pes anserinus insertion to the most medial part of knee joint as shown (Fig. 1b).

The incision penetrated the skin and deep to the bone in knee extension position, then the skin was peeled out. The upper edge of the dissection was 5cm above the proximal part of patella, and the lower edge was 5cm below the tibial tubercle. The skin was removed from the medial to lateral side in one flap. Removal of the skin was performed very carefully in the specimens due to the nerve running through the superficial tissue around the infrapatellar region.

A second deeper superficial incision was done along the lines of the primary incision to access the gracilis and sartorius muscle. The superficial fascia was removed meticulously, causing a large proportion of the IPBSN to be located within the layer of superficial fascia or emerge on the posterior side of the sartorius muscle. Some variations of the saphenous nerve were found and run along the femoral artery and underneath the sartorius muscle. The saphenous nerve started to branch at the sartorius muscle and emerged from underneath the sartorius muscle to supply the anterolateral part of leg.

**Fig 2:**
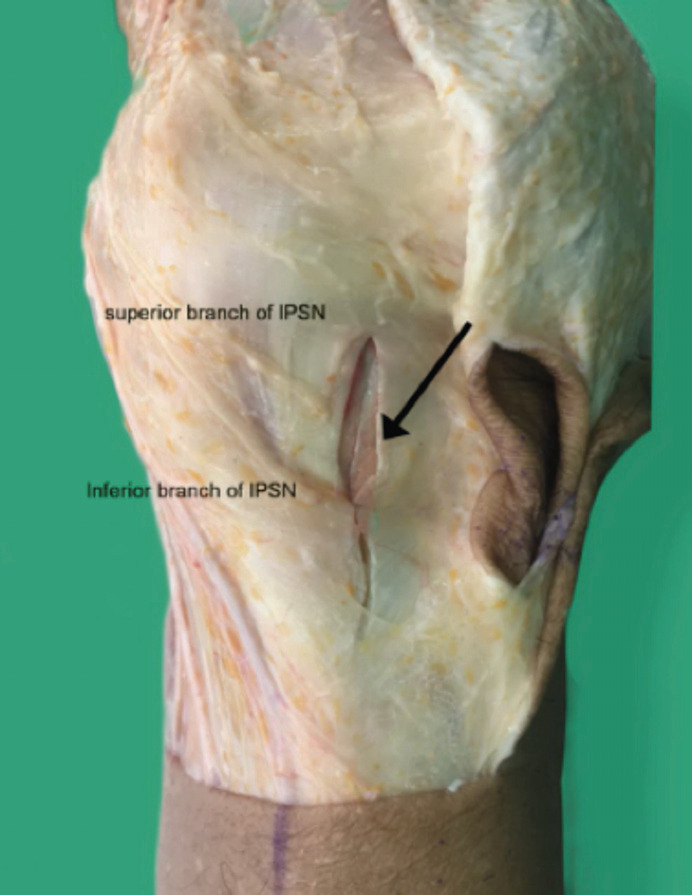
This picture shows the superior and inferior branch of IPBSN injuries; Black arrow is the vertical incision.

Meticulous exploration was performed from the skin to the IPBSN around incision zone. If the discontinuity of the nerve was found, it would be classified as IPBSN injury (Fig. 2).

**Fig 3:**
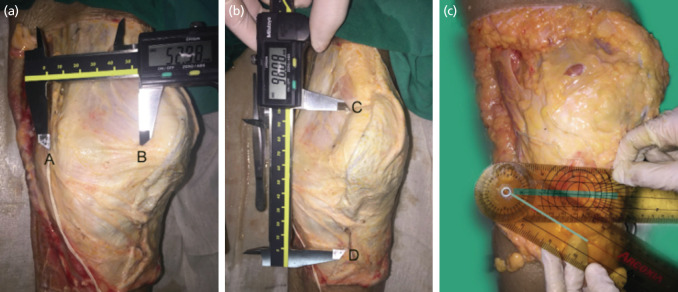
(a) Horizontal distance was shown; A is the superior part of IPBSN at the same level as the mid point of the patella, B is the medial border. (b) Vertical distance was shown; C is the upper pole of patella. C is the superior branch of IPBSN. (c) Declination angle is the intersection between a horizontal line (thick green line) that project from tibial tubercle to the medial side of knee.

Digital Vernier caliper was used to measure distance (centimetre) and goniometer was used to measure the angle(degree). Horizontal distance (centimetre) is the distance from the mid point of patella extending medially in a horizontal line, to the superior branch of IPBSN was shown (Fig. 3a). Vertical distance is the vertical length from the most upper pole of patella to the superior branch of IPBSN which is located midway between the medial aspect of the tibial tuberosity and the medial aspect of the tibia. This vertical distance (centimetre) was shown (Fig. 3b). Declination angle is the intersection between a horizontal line which project from the tibial tubercle to the medial side of knee and the nerve line (the most proximal nerve branch of IPBSN) was shown (Fig. 3c).

In statistical analysis, the data of IPBSN injury between two groups were analysed with the Fisher’s exact test. p<0.005 was considered statistically significant. All analyses were performed using the SPSS 21.0. For the anatomy of IPBSN, we used mean±SD for results.

## Results

The data of cadavers are female 28 (63.6%), male 16(36.4%), and the left side is equal to the right side, as shown in Table I. The risks of IPBSN injury in oblique group are 2 from 22 knees (9.1%) compared with 12 knees (54.5%) in vertical group (P=0.001) as shown in Table II.

**Table I:** Baseline characteristics

	Vertical N (%)	Oblique N (%)
Gender		
Female	15 (34.1)	13 (29.5)
Male	7 (15.9)	9 (20.5)
Total = 44		
Side of knee		
Left	22	22
Right	22	22
Total = 44		

**Table II:** The IPBSN injury between vertical and oblique incision

	Vertical N (%)	Oblique N (%)	P value
IPBSN injury	12 (54.5)	2 (9.1)	.001

These anatomic studies show the most common number of branch(es) found, is one branch, the horizontal distance range from 2.6cm to 8.5cm (average 5.7±1.6), the vertical distance ranged from 4.4cm to 12.6cm (average 7.6±1.9) and the declination angle ranged from 6° to 87° (average 34.7±24.3). There are no differences in the morphological anatomy between male and female, as shown in Table III.

**Table III:** The anatomic study of IPBSN in number of branch(es), horizontal and vertical distance, and decline angle

	Result	P Value
Number of branch(es)		
1	27 (61.4%)	
2	15 (34.1%)	
3	2 (4.5%)	
Horizontal distance		
Range	2.6-8.5cm	0.96
Mean±SD	5.7±1.6	
Female	5.6±1.61	
Male	5.7±1.90	
Vertical distance		
Range	4.4-12.6cm	0.12
Mean±SD	7.6±1.9	
Female	7.9±1.9	
Male	7.0±1.5	
Declination Angle		
Range	6° - 87°	0.97
Mean±SD	34.7±24.3	
Female	33.79±24.80	
Male	35.61±23.60	

## Discussion

Many anatomic studies show variations of this nerve and are related with nerve injury during knee surgery^1,3-5^. Lumsden reported a mean distance from the superior patellar pole of 9.8 cm (7.5cm –14.3cm)^9^. Worth described a mean distance of 10cm from the medial femoral epicondyle^10^. Arthornthurasook *et al* studied IPBSN in all 230 cadavers, they classified the nerve into 4 types according to its relationship to the sartorious muscle: posterior, penetrating, parallel, and anterior^11^.

Painful neuromas due to the IPBSN injury while placing the anteromedial arthroscopic portal or medial side of knee surgery is one of the complications. So, the prevention to IPBSN injury is important^12^.

The IPBSN is the cutaneous sensation of the anteromedial aspect of the knee. The location is highly variable, and no definite safe zone could be identified. Currently, no papers studied the relationship between skin incision and IPBSN injury in medial opening wedge HTO. The advantage of an oblique incision is a small incision that uses as a minimally invasive technique has a lower risk of complication13. In the oblique incision group, the direction of incision is almost parallel to the nerve, so the risk of IPBSN injury is lower (9.1%) than the vertical incision which potentially can cross the nerve (54.5%) in our study. We recommend that the oblique skin incision should be done in less than 45°. To avoid IPBSN injury, one should be careful when the incision is anywhere within 7cm below the joint line.

The limitations of the study are that there are no related clinical outcomes such as the severity of symptoms and how the symptoms affect the daily activities. Therefore, further studies are needed.

## Conclusion

The risk of the IPBSN injury in the oblique skin incision may be less than the vertical incision in the medial opening wedge HTO.
